# Altitudinal Variation in Trade‐Off Strategies of the Plant Economics Spectrum of *Pinus massoniana* Plantations in Subtropical Mountains

**DOI:** 10.1002/ece3.73977

**Published:** 2026-07-10

**Authors:** Kun Nie, Ming Xu, Jian Zhang

**Affiliations:** ^1^ College of Life Sciences, Guizhou Key Laboratory of Agricultural Microbiology Guizhou University Guiyang China; ^2^ National Key Laboratory of Green Pesticide, Key Laboratory of Green Pesticide and Agricultural Bioengineering, Ministry of Education Center for R&D of Fine Chemicals of Guizhou University Guiyang China

**Keywords:** altitude, *Pinus massoniana*, plant economics spectrum, trade‐off

## Abstract

Elevational gradients are natural laboratories for plant ecological strategies, much as altitudinal shifts in tree composition have advanced understanding of forest assembly and dynamics. Yet, functional trait‐based investigations into the ecological strategies of *P. massoniana* plantations communities along subtropical altitudinal gradients remain remarkably scarce. This knowledge gap hinders science‐based management of these forests in subtropical montane habitats. This study investigated pure *P. massoniana* plantations across four altitude gradients (1200–1500 m) in the subtropical mountain forest ecosystem. We quantified functional traits of different organs (leaves and roots) in *P. massoniana* and understory shrubs to decipher their coordinated variations with altitude. Our study revealed divergent trade‐offs in the leaf economics spectrum (LES), root economics spectrum (RES), and whole‐plant economics spectrum (WES) of woody plants in *P. massoniana* plantations along subtropical montane altitudinal gradients. *P. massoniana* showed a shift from acquisitive to conservative trait patterns with increasing elevation, whereas understory shrubs exhibited opposite strategies. Significant correlations emerged between leaf and root traits within the *P. massoniana* plantations communities. The plant economics spectrum showed significant consistency across different organs of species, and there were significant positive correlations among LES, RES, and WES. The divergent altitude‐related ecological adaptation strategies observed between *P. massoniana* and shrubs likely reflect shrubs' heightened sensitivity to microenvironments (e.g., canopy effects) and niche complementarity, although this interpretation remains speculative and requires further research. Overall, there is a consistent resource trade‐off strategy among different organs of plants in *P. massoniana* plantations in subtropical mountainous areas to respond to the direct or indirect effects of altitude. This study provides a theoretical basis at the functional trait level for the adaptive management of *P. massoniana* plantations in subtropical montane regions.

## Graphical abstract

This study investigated the resource‐use strategies of trees and understory shrubs along an altitudinal gradient in subtropical *Pinus massoniana* plantations. It revealed that *P. massoniana* shifts from an acquisitive to a conservative strategy with increasing elevation, while understory shrubs show the opposite pattern. Significant coordination was found between leaf and root economic spectra, highlighting differential adaptive mechanisms among plant life forms in response to environmental heterogeneity.

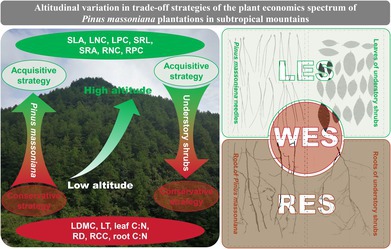

## 1 Introduction

Plant functional traits serve as a critical bridge linking plants to their environments, as they independently or synergistically characterize plant‐ and ecosystem‐level responses to environmental changes, thereby elucidating plant survival strategies and distribution patterns (Diaz et al. 2016; Lavorel 2013; Reich 2014; Reich et al. 2003). With the gradient change of environmental conditions, plant traits often show a trend of inter‐relationship and co‐evolution, which can further reflect the trait change strategies and resource utilization trade‐off strategies of plants at different organizational levels (from organs to species), thus forming the plant economics spectrum (PES) (Bai et al. 2015; Diaz et al. 2016; Reich 2014). As the most prevalent and widely recognized PES, the leaf economics spectrum (LES) reflects trade‐off strategies between resource acquisition and utilization in plants across environments (Reich 2014; Wright et al. 2004). For instance, plants exhibit different resource utilization strategies on the LES under different degrees of drought or along different altitude gradients (Asner et al. 2016; Bai et al. 2015; Silva et al. 2023; Wright et al. 2004). Disturbance and stress gradients can also push plants towards opposite ends of the LES. For example, grazing stress shifts herbaceous plants from acquisitive to conservative strategies (Zhou et al. 2024), fire disturbance strongly impacts the LES of Mediterranean shrublands (Saura‐Mas et al. 2009), and the land use intensity is closely related to the LES (Lienin and Kleyer 2011). Beyond leaves, roots and whole plants also display resource trade‐off spectra—the root economics spectrum (RES) and whole‐plant economics spectrum (WES) (Li et al. 2021; Liese et al. 2017; Roumet et al. 2016). For instance, Stipa species exhibit acquisitive RES and WES in deserts and typical steppes but conservative spectra in meadow steppes (Yang et al. 2024). Similarly, increasing grazing intensity shifts herbaceous RES from conservative to acquisitive (Zhou et al. 2024), and thinning drives *Cunninghamia lanceolata* from the acquisitive towards the conservative end of the RES (Wang, Liu, et al. 2021). Thus, PES across plant organs to whole plants are closely linked to environmental conditions. Investigating variation patterns of PES along environmental gradients enhances understanding of ecological principles and adaptation mechanisms underlying plant responses to environmental changes, thereby providing scientific foundations for ecosystem management and restoration.

In forest ecosystems, variations in plant functional traits and environmental factors collectively determine ecosystem functioning (Chiang et al. 2016; Prado‐Junior et al. 2016). Concurrently, environmental factors are critical drivers of individual plant traits or the PES (Blanchard et al. 2019; Cheng et al. 2022; Vanneste et al. 2019; Wang, Wen, et al. 2021). Therefore, elucidating the relationship between plant functional traits—particularly PES reflecting resource utilization strategies—and environmental factors is essential for understanding forest ecosystem functioning and guiding ecological management (Pérez‐Harguindeguy et al. 2013). Especially in the subtropical mountain forest ecosystem with high environmental heterogeneity and fragile habitat in the karst region of southwest China, the change of environmental factors may have an important impact on plant growth and resource utilization strategies (Li et al. 2013; Ma et al. 2020). However, at present, studies on plant functional traits in subtropical mountain forest ecosystems mainly focus on the following aspects: the internal relationships and differences among traits (Bai et al. 2024; Li et al. 2024), the changing patterns of traits along environmental gradients (Zhao et al. 2016), the influencing factors of traits (Qin and Shangguan 2019; Yang, Gou, et al. 2021), and the impacts of plants on biodiversity, community composition, or ecosystem functions based on functional traits (Miao et al. 2022; Xu et al. 2024; Zhou et al. 2021; Zou et al. 2024). Besides, most studies focus on leaf traits, with limited attention to how PES responds to environmental heterogeneity in forest ecosystems, particularly within conspecific populations or homogeneous forest communities. Consequently, our understanding of resource utilization strategies adopted by plants in this region to adapt to environmental gradients remains incomplete.

*Pinus massoniana* is frequently employed as a pioneer species in vegetation restoration due to its robust environmental adaptability, rapid biomass accumulation rates, and exceptional tolerance to drought and nutrient‐poor soils (Nie et al. 2023; Zhou 2001). Extensive monoculture plantations and conifer‐broadleaf mixed forests dominated by *P. massoniana* not only contribute significantly to regional forestry economies but also play vital roles in ecosystem construction (Nie et al. 2023; Tang et al. 2024). However, as a heliophilic and thermophilic species, *P. massoniana* exhibits heightened sensitivity to thermal fluctuations. Elevational increases alter light and hydrothermal conditions, indirectly impairing its growth, with its upper elevational distribution limit in subtropical mountains approximating 1300 m (Zhou 2001). Given that high‐elevation environments commonly drive conifers towards conservative resource‐use strategies (Reich 2014), we anticipated a similar shift in *P. massoniana* . Currently, studies investigating how altitudinal gradients influence ecological strategies of *P. massoniana* plantation communities through plant functional trait approaches remain scarce, hindering science‐based management of these forests in subtropical montane habitats. Consequently, elucidating elevational resource‐use strategies in *P. massoniana* plantations—particularly adaptive divergences beyond its upper altitude distributional limit—is critical for understanding plant functionality and ecosystem processes in the subtropical mountain forest ecosystem.

Our study quantified leaf and root morphological and nutrient traits of *P. massoniana* and 13 co‐occurring understory shrub species across altitudinal gradients in subtropical montane ecosystems of southwestern China. We specifically addressed four interrelated questions: (1) whether altitudinal economics spectrum exists in leaf, root, and whole‐plant traits of *P. massoniana* plantations; (2) if such PES emerge, what resource trade‐off patterns characterize its altitudinal variation—and, given that high‐elevation environments commonly drive conifers towards conservative resource‐use strategies (Reich 2014), whether *P. massoniana* exhibits a similar shift; (3) whether, owing to differences in life form, *P. massoniana* and understory shrubs display distinct altitudinal patterns in their resource‐use strategies; and (4) whether resource‐use strategies are consistent across‐organs. By integrating multi‐trait analyses at leaf, root, and whole‐plant levels, this work systematically evaluates how subtropical montane plants coordinate resource allocation under altitudinal constraints, bridging functional trait plasticity with ecosystem‐scale adaptation mechanisms.

## 2 Methods

### 2.1 Study Area

**FIGURE 1:**
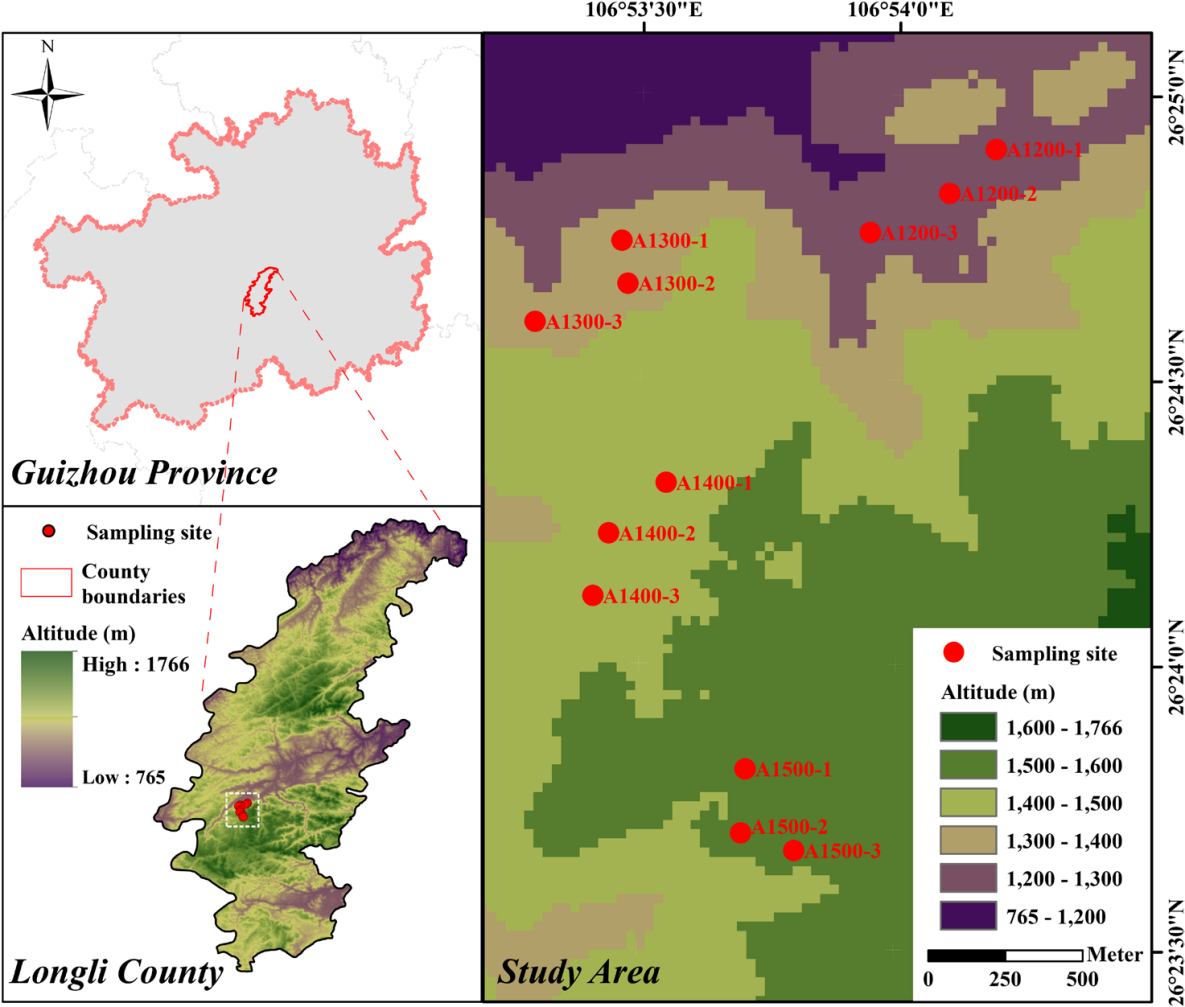
Location of the study area and the sampling plot.

Longli County, located in the central part of Guizhou Province, is characterized by mountainous terrain and karst topography. It has coordinates ranging from 26°21′ to 26°41′E and 106°51′ to 107°11′N, with altitude ranges from 765 to 1766 m a.s.l. (Figure 1). The predominant terrestrial vegetation in this area is evergreen broad‐leaved forest, while areas with limestone soil support coniferous and needle‐ and broad‐leaf mixed forest, typically found in less‐disturbed areas. The forests are mainly composed of *P. massoniana* , with secondary species including *Quercus fabri* and *C. lanceolata* (Nie et al. 2023).

The predominant climate is a subtropical monsoon humid climate, with an average annual precipitation around 1100 mm. The mean annual temperature is 15.0°C, with January being the coldest month at 4.8°C, and July the warmest at 23.5°C. The average annual sunshine duration is approximately 1160 h (Luo et al. 2019; Nie et al. 2023).

### 2.2 Plot Design and Sampling

Four altitudes were selected at intervals of 100 m from 1200 to 1500 m a.s.l at Longli Forest Farm. This specific gradient was determined by the natural distribution and silvicultural history of *P. massoniana* in the region (Nie et al. 2023). Within the heterogeneous karst landscape, extensive, contiguous, and even‐aged pure *P. massoniana* plantations covering a larger altitude span are exceptionally rare. The selected gradient represents the maximum contiguous altitude range of homogeneous *P. massoniana* monoculture available for controlled study (Nie et al. 2023). To isolate the effect of altitude, all sampling plots were established on comparable topographic settings within the same contiguous forest tract (Table S1), rather than comparing stands that, while having a greater total altitude difference, are geographically separate and differ in other critical environmental factors. This design ensures that the observed functional trait variations can be most parsimoniously attributed to the altitudinal gradient per se.

At each altitude, three independent replicate sampling plots (20 × 20 m) with relatively consistent stand ages were established. The developmental timeline of the *P. massoniana* forest between 1200 to 1500 m a.s.l was uniform, with an average forest age of 40–53 years (Figure 1 and Table S1). Basic environmental information for each sampling plot was recorded, and a vegetation survey was conducted by establishing five shrub investigation quadrats within each sampling plot. From each sampling plot, leaf and root samples were taken. The collection of leaf and root samples was performed following a previously described protocol (Pérez‐Harguindeguy et al. 2013). In brief, in each plot, three healthy and evenly growing *P. massoniana* were chosen. Sun‐exposed healthy branches were collected from each tree's crown in four directions (east, west, south, north), and second‐year needles (those grown the previous year and crucial for photosynthesis during the growing season) were separated based on their attachment position, bud scale scars, and needle color. The second‐year needles collected from all four directions are thoroughly mixed to form a composite sample. Simultaneously, collect roots of these 3 *P. massoniana* . Since the fine roots of *P. massoniana* are mainly distributed in the 0–20 cm soil layer (Cheng et al. 2012; Song et al. 2020), and in karst topography areas, plant roots are mainly distributed in the 0–10 cm soil layer (Zhang, He, et al. 2020), the root sampling depth selected in this study is 0–20 cm. The use of soil cubes (20 × 20 × 20 cm) follows the classical “monolith” method, which is widely recommended for capturing intact fine root systems and obtaining reliable root trait data (Freschet et al. 2021). Collect root samples from 8000 cm^3^ soil‐cube (a cube with a side length of 20 cm) in areas 1 m away from the trunk in the southeast by 45°, northeast by 75° and southwest by 15° directions of *P. massoniana* . These three spatially independent sampling directions per tree reduce positional bias and ensure representative root collection across the root zone. In a pure *P. massoniana* plantation, the characteristic resinous odor, reddish‐brown color, and straighter morphology of *P. massoniana* roots allow reliable visual‐olfactory separation from shrub and herb roots, a practice supported by root identification guidelines (Rewald et al. 2012). Pick out all live roots from the soil, and separate the root samples of *P. massoniana* and shrubs based on their appearance, color, and odor. Combine the two types of roots picked from the three soil samples of each tree into one sample, respectively. The collection of shrub leaf and root samples followed the protocol used for *P. massoniana* . Based on the quadrat survey data, shrub species for sampling were selected according to the following criteria: frequency of occurrence > 20% across all quadrats, high importance value, and sufficient abundance. Consequently, seven dominant shrub species were ultimately selected: *Rhododendron simsii*, *Quercus dentata* , *Smilax polycolea*, *Castanea seguinii*, *Eurya japonica* , *Clethra delavayi*, and *Rubus corchorifolius* (Table S2 and Figures S1 and S2). Randomly collect more than 20–30 mature leaves from 3 to individuals without obvious shading from each shrub in 4 directions, and collect root samples from 3 directions 0.5 m away from the trunk of shrub. Immediately wrap leaf and root samples with deionized water‐soaked filter paper, place them in self‐sealing bags, and store them in a 4°C insulated cooler for transport back to the laboratory.

### 2.3 Traits Measurements

Needle length (NL), width (NW), and thickness (LT) of *P. massoniana* were measured using a digimatic caliper (Mitutoyo CD‐6ASX, Japan, precision ±0.01 mm) and dial thickness gauge (Peacock G‐7C, Japan, precision ±0.001 mm). For NW and LT, measurements were taken at 1/4, 1/2, and 3/4 positions along the needle length, with mean values calculated. For understory shrubs, LT was measured at three secondary vein‐avoiding locations on both sides of the midrib, averaged per leaf. Leaf area (LA, mm^2^) of shrubs was determined using a flatbed scanner (Epson Perfection V19, Japan) and image analysis software of leaf (LeafArea). Needle LA was calculated via the formula LA = (1 + π/2) × NW × NL, following established methods (He et al. 2020; Zhang et al. 2017). Fresh weight (FW) of fully rehydrated leaves was measured after surface moisture removal. Samples were then oven‐dried at 60°C to constant mass (~48 h) for dry weight (LDW) determination. Leaf dry matter content (LDMC = LDW/FW) and specific leaf area (SLA = LA/LDW) were derived from these measurements. Fine roots were scanned using a root scanner (Epson Perfection V850 Pro, Japan), with root diameter (RD), root surface area (RSA), and root length (RL) analyzed via WinRHIZO Pro 2009b software. Following scanning, roots were oven‐dried to constant mass for dry weight (RDW) measurement. Specific root length (SRL = RL/RDW) and specific root area (SRA = RSA/RDW) were subsequently calculated. Community‐weighted mean trait values (CWM) were calculated for understory shrubs of each plot based on the relative contribution of the species to the community (Lavorel et al. 2008).

$$\mathrm{CWM}=\sum_{i=1}^{n}P_{i}\times {\text{trait}}_{i}$$

where *P*_*i*_ means the relative contribution of species *i* to the community for which we use the IV indices, and *trait*_*i*_ is the trait value of species *i*.

For further analysis, dried leaf or root samples were ground using a ball mill and sieved through a 0.25 mm mesh. Elemental contents of the samples were determined using the following methods: Leaf carbon content (LCC) and root carbon content (RCC) were measured via K_2_Cr_2_O_7_‐H_2_SO_4_ oxidation with external heating (Wu and O'Donnell 1997); Leaf nitrogen content (LNC) and root nitrogen content (RNC) were analyzed through Kjeldahl nitrogen determination after H_2_O_2_‐H_2_SO_4_ digestion (NY/T2017‐2011 2011); Leaf phosphorus content (LPC) and root phosphorus content (RPC) were quantified by molybdenum‐antimony anti‐spectrophotometric method following H_2_O_2_‐H_2_SO_4_ digestion (NY/T2017‐2011 2011); Leaf potassium content (LKC) and root potassium content (RKC) were determined using flame atomic absorption spectrophotometry post H_2_O_2_‐H_2_SO_4_ digestion (NY/T2017‐2011 2011). The stoichiometric ratios of leaf carbon to nitrogen (leaf C:*N* = LCC/LNC), leaf nitrogen to phosphorus (leaf N:*P* = LNC/LPC), root carbon to nitrogen (root C:N = RCC/RNC), and root nitrogen to phosphorus (root N:P = RNC/RPC) were also calculated.

### 2.4 Data Analysis

All statistical analyses and mapping were conducted using R 4.2.1 software. Differences in plant functional traits along altitudinal gradients were analyzed using Wilcoxon tests from the *rstatix* package, while univariate elevational trends of functional traits were calculated using the *dplyr* package.

To determine the correlation between leaf and root traits, the Pearson correlation coefficients were calculated with the *psych* package. To control the false discovery rate (FDR) due to multiple comparisons, the Benjamini‐Hochberg procedure was applied at *q* < 0.05 (Benjamini and Hochberg 1995). Subsequently, the correlation networks were constructed using the *igraph* package. In the network, the correlation coefficients were used to weight the connecting lines. The thicker the connecting line, the stronger the correlation between the two traits. The size of the connecting node represents the connectivity of the trait. The larger the connecting node, the higher the connectivity (the number of connections between one trait and other traits). Meanwhile, Multiple Factor Analysis (MFA) was performed using the *FactoMineR* package to validate trait covariation patterns.

To identify potential LES, RES, and WES, principal component analysis (PCA) was conducted separately for leaf, root, and whole‐plant traits using the *FactoMineR* package, with results visualized via the *factoextra* package. Bivariate correlations between individual traits and the first two principal components (PC1 and PC2) were assessed using Pearson's *r* to quantify trait contributions to and preferences for these axes. Wilcoxon tests were employed to compare PC1 scores (PC1S) of leaf, root, and whole‐plant traits across altitudinal zones and growth forms.

Finally, to test the degree of association among LES, RES, and WES, we analyzed the bivariate correlations among leaf PC1, root PC1, and whole‐plant PC1 using the standardized major axis (SMA) regression method (Li et al. 2022). The scaling equation *y* = βxα is used to evaluate the bivariate trait relationship. By performing a logarithmic transformation on this equation, a linear form lg (y) = lg (β) + αlg (x) is obtained, where y and x represent the traits of interest, α represents the log–log slope, and β represents the y‐intercept of the linear regression curve (Niklas 1994). When *α* = 1, the two variables y and x are isometrically related (i.e., a one‐to‐one scaling relationship exists), and when *α* > 1 or *α* < 1, the two variables y and x are non‐isometric (allometrically) related to one another (Penny 1992). The *smatr* package is used to determine the slopes of the three bivariate relationships: leaf PC1 vs. root PC1, whole‐plant PC1 vs. leaf PC1, and whole‐plant PC1 vs. root PC1. The *ggplot2* package is used to visualize these relationships.

## 3 Results

### 3.1 Responses of Plant Functional Traits in *P. massoniana* Forests to Altitude Gradients

**FIGURE 2:**
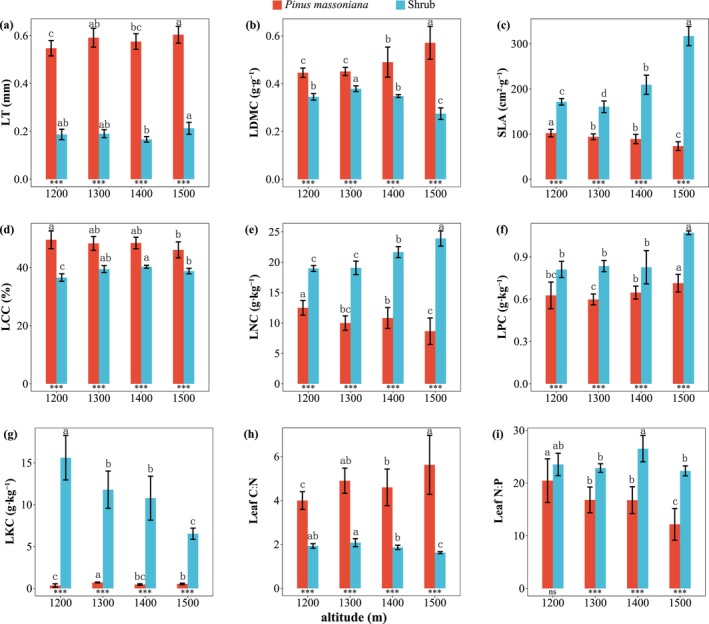
Characteristics of leaf traits of *P. massoniana* and understory shrubs along different altitude gradients. (a) Leaf thickness (LT), (b) Leaf dry matter content (LDMC), (c) Specific leaf area (SLA), (d) Leaf carbon content (LCC), (e) Leaf nitrogen content (LNC), (f) Leaf phosphorus content (LPC), (g) Leaf potassium content (LKC), (h) Stoichiometric ratios of leaf carbon to nitrogen (Leaf C:N), and (i) Stoichiometric ratios of leaf nitrogen to phosphorus (Leaf N:P). Red bars represent *P. massoniana* , blue bars represent understory shrubs. Different lowercase letters indicate that there are significant differences (*p* < 0.05) in the traits of *P. massoniana* or shrubs among different altitudes. Asterisks indicate that there are significant differences in the traits between *P. massoniana* and shrubs at the same altitude (***, *p* ≤ 0.001; ns, *p* > 0.05).

**FIGURE 3:**
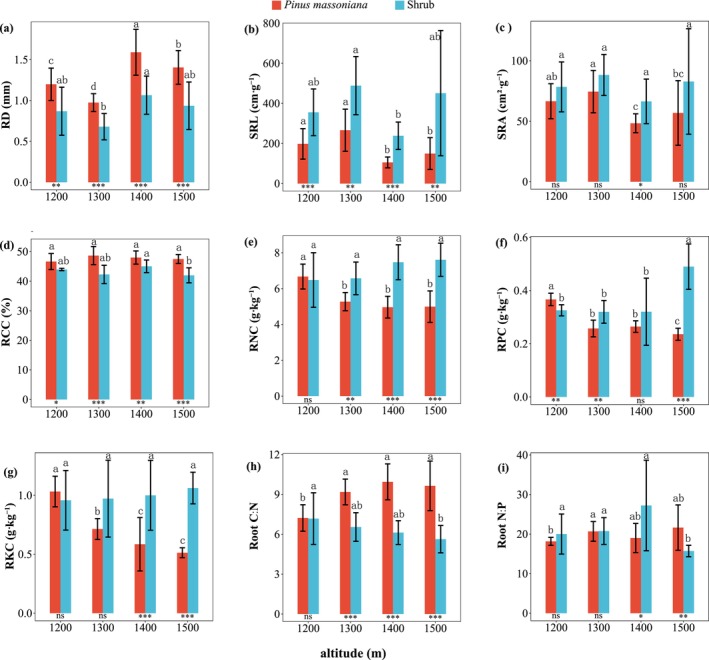
Characteristics of root traits of *P. massoniana* and understory shrubs along different altitude gradients. (a) Root diameter (RD), (b) Specific root length (SRL), (c) Specific root area (SRA), (d) Root carbon content (RCC), (e) Root nitrogen content (RNC), (f) Root phosphorus content (RPC), (g) Root potassium content (RKC), (h) Stoichiometric ratios of root carbon to nitrogen (Root C:N), and (i) Stoichiometric ratios of root nitrogen to phosphorus (Root N:P). Red bars represent *P. massoniana* , blue bars represent understory shrubs. Different lowercase letters indicate that there are significant differences (*p* < 0.05) in the traits of *P. massoniana* or shrubs among different altitudes. Asterisks indicate that there are significant differences in the traits between *P. massoniana* and shrubs at the same altitude (***, *p* ≤ 0.001; **, *p* ≤ 0.01; *, *p* ≤ 0.05; ns, *p* > 0.05).

Given that the composition of these dominant shrub species was largely consistent across the entire altitudinal gradient (Figure S2), we compared the CWM of shrub traits among altitudes. The results showed that both the leaf and root traits of *P. massoniana* and understory shrubs changed markedly along the altitude gradient (Figures 2 and 3). Except for RCC, all other traits of *P. massoniana* exhibited significant differences along the altitude gradient (*p* < 0.05). Specifically, LT, LDMC, LPC, leaf C:N, RD, root C:N, and root N:P increased significantly with increasing altitude, whereas SLA, LCC, LNC, leaf N:P, SRL, SRA, RNC, RPC, and RKC decreased significantly as the altitude increased (Figure S3, *p* < 0.05). Except for SRA, RNC, and RKC, all other functional traits of understory shrubs showed significant differences along the altitude gradient (*p* < 0.05). Specifically, SLA, LCC, LNC, LPC, RNC, and RPC increased significantly with increasing altitude, while LDMC, LKC, leaf C:N, and root C:N decreased significantly with altitude. The remaining traits showed no clear trend along the altitude gradient (Figure S3, *p* < 0.05). In addition, there were significant differences in leaf functional traits between trees and shrubs at the same altitude. The LT, LDMC, LCC, and leaf C:N of *P. massoniana* were significantly higher than those of shrub plants, while SLA, LNC, LPC, LKC, and leaf N:P were significantly lower than those of shrubs (*p* < 0.001, Figure 2a–i). Regarding root traits, there were significant differences in RD, SRL, and RCC across all altitudes, while the remaining traits did not show significant differences at all altitudes (*p* < 0.001, Figure 3a–i).

### 3.2 Correlation Between Leaf Traits and Root Traits

**FIGURE 4:**
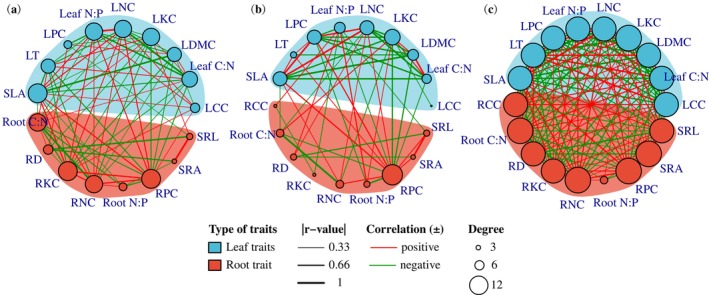
The trait correlation network of *P. massoniana* (a), shrub (b), and *P. massoniana* and shrub (c). Different colors represent different types of plant organs. Cerulean Blue represents leaf traits. Coral Red represents root traits. Node size shows connectedness. The number in parentheses after the abbreviation of a trait represents the total number of connections between that trait and other traits. Red and green edges represent positive and negative correlations (*p* < 0.05), respectively. Connecting lines of different widths represent different degrees of correlation. When *p* < 0.05, we used 1.5 pt. wide connecting lines. When *p* < 0.01, we used 2.5 pt. wide connecting lines. When *p* < 0.001, we used 4 pt. wide connecting lines.

In *P. massoniana* , shrub, and all woody plants, most functional traits of leaves and roots were significantly correlated with one another (*p* < 0.05, Figure 4 and Figure S4). The traits with the highest connectivity in *P. massoniana* were SLA, RKC, and RPC, followed by LKC, LNC, leaf N:P, root C:N, RNC, leaf C:N, LDMC, and LT (Figure 4a). The trait with the highest connectivity in shrub species was RPC, followed by LKC, LNC, LPC, and SLA. Among these traits, there was a strong correlation between leaf C:N and other functional traits (Figure 4b). For all woody plants in the *P. massoniana* forest (i.e., *P. massoniana* and shrubs), with the exception of root N:P, the remaining functional traits showed relatively high connectivity. Notably, there was a strong correlation between leaf C:N and other functional traits (Figure 4c). While these correlations statistically link leaf and root traits, they do not directly demonstrate coordinated adaptation mechanisms; nevertheless, they tentatively suggest that trait integration may contribute to plant responses to altitudinal environmental changes.

### 3.3 Altitudinal and Growth Form Differentiation of Plant Resource Utilization Strategies in *P. massoniana* Forests: Analysis Based on the Leaf, Root, and Whole‐Plant Economic Spectrum of Multi‐Trait Synergy and Trade‐Off

**FIGURE 5:**
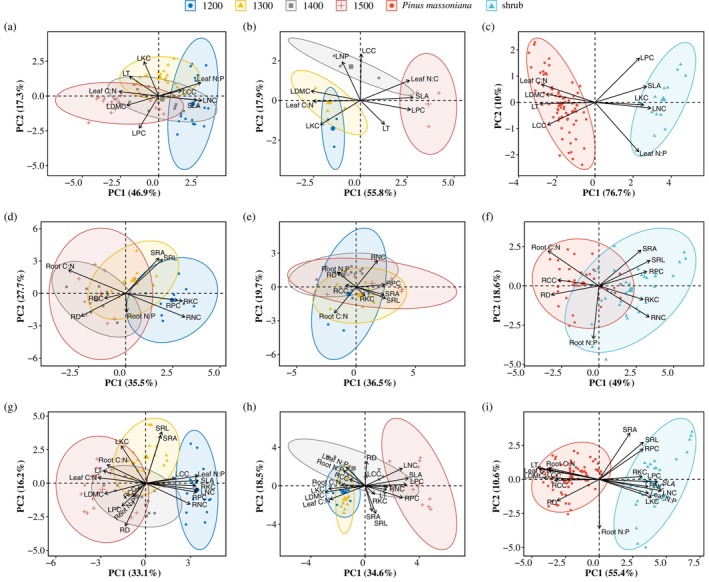
Principal component analysis (PCA) of plant functional traits in *P. massoniana* plantations across elevation gradients. (a) Leaf traits and leaf economic spectrum (LES) of *P. massoniana* . (b) Leaf traits and LES of understory shrubs. (c) Leaf traits and LES by plant growth form. (d) Root traits and root economic spectrum (RES) of *P. massoniana* . (e) Root traits and RES of understory shrubs. (f) Root traits and RES by plant growth form. (g) Whole‐plant traits and whole‐plant economic spectrum (WES) of *P. massoniana* . (h) Whole‐plant traits and WES of understory shrubs. (i) Whole‐plant traits and WES by plant growth form.

**FIGURE 6:**
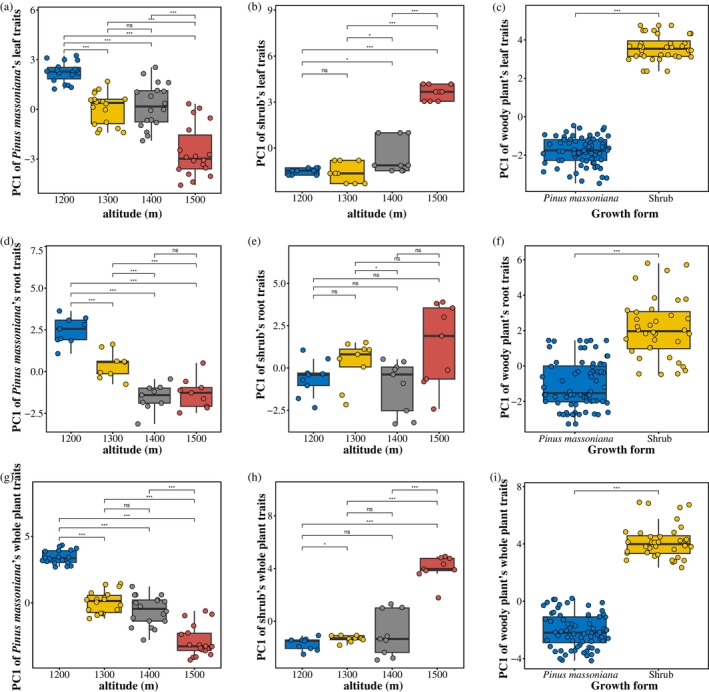
Differences in PC1 score values of leaf, root and whole‐plant functional traits among altitude gradients and different growth forms via nonparametric test. (a) PC1 scores of leaf traits for *P. massoniana* along four altitudes, (b) PC1 scores of leaf traits for understory shrubs along four altitudes, (c) Inter‐group comparison of leaf trait PC1 scores between *P. massoniana* and understory shrubs (different growth forms), (d) PC1 scores of root traits for *P. massoniana* along four altitudes, (e) PC1 scores of root traits for understory shrubs along four altitudes, (f) Inter‐group comparison of root trait PC1 scores between *P. massoniana* and understory shrubs, (g) PC1 scores of whole‐plant traits for *P. massoniana* along four altitudes, (h) PC1 scores of whole‐plant traits for understory shrubs along four altitudes, (i) Inter‐group comparison of whole‐plant trait PC1 scores between *P. massoniana* and understory shrubs. Different asterisks denote significant differences (***, *p* ≤ 0.001; *, *p* ≤ 0.05; ns, *p* > 0.05).

According to the PES theory (Wright et al. 2004; Reich 2014), acquisitive strategies are indicated by trait combinations that promote rapid resource capture (e.g., high SLA, high LNC, low LDMC), whereas conservative strategies are indicated by the opposite combinations. In this study, the coordinated and trade‐off variations of multiple traits along the principal component axes revealed the resource utilization strategies of leaves and roots across altitudinal gradients and growth forms. Accordingly, the traits representing resource acquisition or resource conservation strategies were located on both sides of the first principal component (PC1) axis, defining the LES, RES, and WES (Figure 5). PC1 and second principal component (PC2) accounted for 46.9% and 17.3% of the variation in leaf traits of *P. massoniana* , respectively (Figure 5a). All leaf traits were significantly correlated with the PC1 (Table S3). Among them, LNC, leaf N:P, leaf C:N, and SLA made substantial contributions to the PC1 (Figure S5a). Along the PC1 axis, leaf traits associated with the conservation strategy (leaf C:N, LDMC, and LT) decreased, whereas those related to the acquisition strategy (LNC and SLA) increased, thus defining the LES of *P. massoniana* . As the altitudinal gradient increased, the leaves of *P. massoniana* shifted from the acquisition strategy to the conservation strategy (Figure 5a). Additionally, the first principal component score (PC1S) values of *P. massoniana* at higher altitudes were significantly lower than those at lower altitudes (*p* < 0.001, Figure 6a).

PC1 and PC2 accounted for 55.8% and 17.9% of the variation in leaf traits of shrub species, respectively (Figure 5b). With the exception of LCC, all other leaf traits were significantly correlated with PC1 (Table S3). Among them, SLA, LPC, LDMC, LNC, and leaf C:N made significant contributions to PC1 (Figure S5b). Along the PC1 axis, leaf traits representing the acquisition strategy (SLA, LPC, and LNC) increased, whereas those representing the conservation strategy (LDMC and leaf C:N) decreased, defining the LES of shrubs. As the altitudinal gradient increased, the leaf traits of shrub plants shifted from the conservation strategy to the acquisition strategy (Figure 5b). Additionally, the PC1S values of shrub species at 1400 m and 1500 m were significantly higher than those at 1200 m and 1300 m (*p* < 0.05, Figure 6b).

In terms of growth form, PC1 and PC2 accounted for 76.7% and 10.0% of the variation in leaf traits of plants, respectively (Figure 5c). All leaf traits were significantly correlated with the PC1 (Table S3). Among them, LNC, LT, leaf C:N, and SLA made significant contributions to the PC1 (Figure S5c). Along the PC1 axis, leaf traits representing the acquisition strategy (LNC, LPC, and SLA) increased, whereas those representing the conservation strategy (LCC, leaf C:N, LDMC, and LT) decreased, defining the LES of woody plants in *P. massoniana* plantations. All *P. massoniana* (trees) were on the conservative side of LES, while all shrub plants were on the acquisition side of LES (Figure 5c). Additionally, the PC1S values of *P. massoniana* were significantly lower than those of shrub species (*p* < 0.001, Figure 6c).

In terms of root systems, PC1 and PC2 accounted for 35.5% and 27.7% of the variation in root traits of *P. massoniana* , respectively (Figure 5d). Apart from RCC and root N:P, the other root traits were significantly correlated with PC1 (Table S3). Among them, RNC, root C:N, RKC, RPC, and RD made significant contributions to PC1 (Figure S5d). Along the PC1 axis, the root traits representing the acquisitive strategy (SRL, SRA, RNC, and RPC) increased, whereas those representing the conservative strategy (RD, RCC, and root C:N) decreased, which defined the RES of *P. massoniana* . *P. massoniana* at high altitude was on the side of the conservative strategy, while those at low altitude were on the acquisitive side (Figure 5d). The PC1S of *P. massoniana* at low altitude was significantly higher than that at high altitude (*p* < 0.001, Figure 6d).

PC1 and PC2 accounted for 36.5% and 19.7% of the variation in root traits of shrub plants, respectively (Figure 5e). Except for RCC and RKC, the remaining root traits were significantly correlated with the PC1 (Table S3), among which SRL, RPC, and SRA made important contributions to the PC1 (Figure S5e). Along the PC1 axis, root traits representing the acquisitive strategy (SRL, SRA, RNC, and RPC) increased, while root traits representing the conservative strategy (RD, RCC, and root C:N) decreased, defining the RES of shrubs; shrub plants at the four altitudes almost overlapped with each other (Figure 5e), and there were hardly any significant differences in root traits among them along the PC1 axis (*p* > 0.05, Figure 6e).

Regarding growth forms, PC1 and PC2 accounted for 49% and 18.6% of the variation in root traits of plants, respectively (Figure 5f). Apart from root N:P, the other root traits made relatively large contributions to the PC1 axis (Table S3). Among them, SRL, root C:N, RNC, RPC, and RD made significant contributions to the PC1 (Figure S5f). Along the PC1 axis, root traits representing the acquisitive strategy (RNC, RPC, SRL, and SRA) increased, while root traits representing the conservative strategy (RD, RCC, and root C:N) decreased, defining the RES of woody plant in *P. massoniana* plantations. Most *P. massoniana* were on the conservative end, while most shrub plants were on the acquisitive end (Figure 5f). Moreover, the PC1S of shrub plants was significantly higher than that of *P. massoniana* (*p* < 0.001, Figure 6f).

For the whole‐plant, PC1 and PC2 explained 33.1% and 16.2% of the whole‐plant trait variation in *P. massoniana* , respectively (Figure 5g). With the exception of RCC and root N:P, other whole‐plant traits showed a significant correlation with the PC1 (Table S3). Among these, LNC, SLA, leaf N:P, RPC, RKC, leaf C:N, RNC, LT, LDMC, and root C:N made significant contributions to the PC1 (Figure S5g). Along the PC1 axis, whole‐plant traits indicative of the acquisitive strategy (e.g., LNC, RNC, RPC, SLA, SRL, and SRA) increased, whereas those representing the conservative strategy (e.g., leaf C:N, LT, LDMC, RD, and root C:N) decreased, thus defining the WES of *P. massoniana* . *P. massoniana* at low altitudes tended towards the acquisitive end, whereas those at high altitudes leaned towards the conservative end (Figure 5g). Additionally, the PC1S value of *P. massoniana* at low altitudes was significantly higher than that at high altitudes (*p* < 0.001, Figure 6g).

PC1 and PC2 accounted for 34.6% and 18.5% of the whole‐plant trait variation in shrub species, respectively (Figure 5h). With the exception of LCC, RKC, RD, SRL, and SRA, other whole‐plant traits had a significant correlation with the PC1 (Table S3). Among these, SLA, LPC, LDMC, RPC, LKC, and leaf C:N made significant contributions to the PC1 (Figure S5h). Along the PC1 axis, whole‐plant traits representing the acquisitive strategy (e.g., LNC, LPC, RNC, RPC, and SLA) increased, while those representing the conservative strategy (e.g., leaf C:N, LDMC, RCC, and root C:N) decreased, defining the WES of shrubs. Shrub species at high altitudes tended towards the acquisitive end, whereas those at low altitudes leaned towards the conservative end (Figure 5h). Additionally, the PC1S value of shrub at high altitudes was significantly higher than that at low altitudes (*p* < 0.05, Figure 6h).

In terms of growth form, PC1 and PC2 accounted for 55.4% and 10.6% of the whole‐plant trait variation in plants, respectively (Figure 5i). Except for root N:P, other whole‐plant traits were significantly correlated with PC1 (Table S3). Among these, LNC, LT, leaf C:N, SLA, LDMC, LKC, LCC, root C:N, leaf N:P, and LPC made significant contributions to PC1 (Figure S5i). Along the PC1 axis, whole‐plant traits indicative of the acquisitive strategy (LNC, LPC, RNC, RPC, SLA, SRL, and SRA) increased, whereas those representing the conservative strategy (LCC, leaf C:N, RCC, root C:N, LT, LDMC, and RD) decreased, defining the WES of woody plants in *P. massoniana* plantations. All *P. massoniana* (trees) tended towards the conservative end, whereas shrub species leaned towards the acquisitive end (Figure 5i). Additionally, the PC1S value of shrub species was significantly higher than that of *P. massoniana* (*p* < 0.001, Figure 6i).

### 3.4 Scaling Relationships Among LES, RES and PES

**FIGURE 7:**
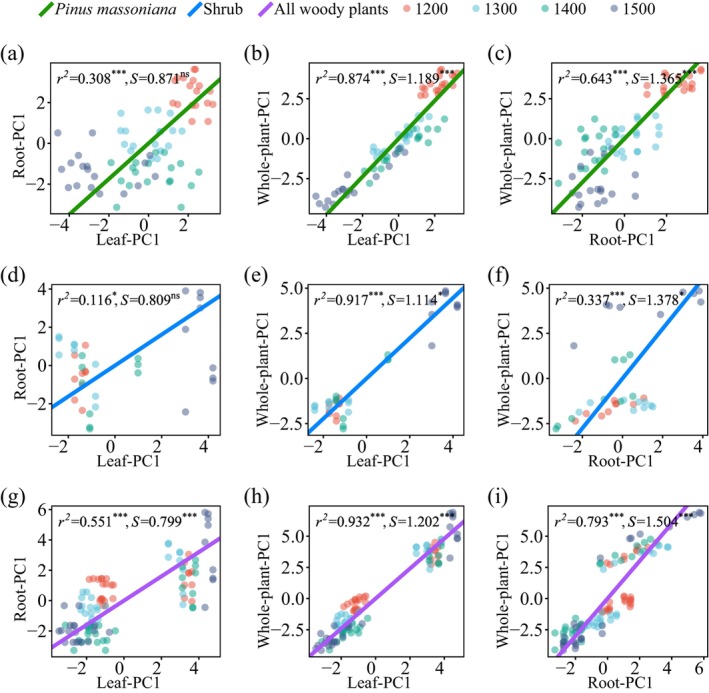
Regression relationships between Leaf‐PC1, Root‐PC1, and Whole‐plant‐PC1 of *P. massoniana*, understory shrubs, and all woody plants in habitats at different altitudes. (a) Leaf‐PC1 vs. Root‐PC1 for *P. massoniana*, (b) Leaf‐PC1 vs. Whole‐plant‐PC1 for *P. massoniana*, (c) Root‐PC1 vs. Whole‐plant‐PC1 for *P. massoniana*, (d) Leaf‐PC1 vs. Root‐PC1 for understory shrubs, (e) Leaf‐PC1 vs. Whole‐plant‐PC1 for understory shrubs, (f) Root‐PC1 vs. Whole‐plant‐PC1 for understory shrubs, (g) Leaf‐PC1 vs. Root‐PC1 for all woodyplants, (h) Leaf‐PC1 vs. Whole‐plant‐PC1 for all woody_plants, (i) Root‐PC1 vs. Whole‐plant‐PC1 for all woody plants. Regression lines were plotted when *p* ≤ 0.05. For each result of standardized major axis, *r* ^2^ represents the squared coelation coefficient (a test statistic), and *S* represents the slope. The asterisks after the number of *r* ^2^ and *S* represent the “*p*‐value of the test of the correlation coefficient against 0” and the “*p*‐value of the test if there a common slope equal to 1”, respectively (***, *p* ≤ 0.001; *, *p* ≤ 0.05; ns, *p* > 0.05).

Whether it is *P. massoniana* , shrub plants or all woody plants in the *P. massoniana* plantations, there are significant positive correlations among the PC1 of leaf, root and whole‐plant traits (Leaf‐PC1, Root‐PC1 and Whole‐plant‐PC1, *p* < 0.05, Figure 7). For *P. massoniana* , the *r* ^2^ between the PC1 of its leaf, root and whole‐plant are 0.308, 0.874 and 0.643 respectively (*p* < 0.001); the common regression slope between Leaf‐PC1 and Root‐PC1 showed no statistical difference from 1, indicating an isometric relationship in which the leaf and root economics spectra shift at the same rate along the altitudinal gradient. However, the common regression slopes between Leaf‐PC1 and Whole‐plant‐PC1, and between Root‐PC1 and Whole‐plant‐PC1 were 1.189 and 1.365, respectively, both significantly greater than 1 (*p* < 0.001, Figure 7a–c), indicating an allometric relationship in which the whole‐plant economics spectrum shifts faster than either the leaf or root spectrum alone along the altitudinal gradient. For shrub plants, the *r* ^2^ between the PC1 of their leaf, root and whole‐plant traits are 0.116, 0.917 and 0.337 respectively (*p* < 0.05); the common regression slope between Leaf‐PC1 and Root‐PC1 showed no statistical difference from 1. Nevertheless, the common regression slopes between Leaf‐PC1 and Whole‐plant‐PC1, and between Root‐PC1 and Whole‐plant‐PC1 were 1.114 and 1.378, respectively, both significantly greater than 1 (*p* < 0.001, Figure 7d–f). For all woody plants, the *r* ^2^ between the PC1 of their leaf, root and whole‐plant traits are 0.551, 0.932 and 0.793 respectively (*p* < 0.001); the common regression slopes are 0.799, 1.202 and 1.504 respectively, which have significant statistical differences from 1 (*p* < 0.001, Figure 7g–i). In the three types of relationships, the regression slopes between Leaf‐PC1 and Root‐PC1 of *P. massoniana* and shrub plants showed no significant statistical difference from 1, indicating an isometric growth relationship between the LES and the RES (Figure 7a,d); however, the regression slopes between Leaf‐PC1 and Whole‐plant‐PC1, and between Root‐PC1 and Whole‐plant‐PC1 of both plant types showed significant statistical differences from 1 (*p* < 0.001), indicating an allometric growth relationship between LES and the WES, as well as between RES and WES (Figure 7b,c,e–i).

## 4 Discussion

### 4.1 Leaf, Root, and Whole‐Plant Economic Spectrum Along Altitudinal Gradients in Subtropical Montane *P. massoniana* Plantations

This study found that at the leaf, root, and whole‐plant levels, traits such as LDMC, LT, leaf C:N, RD, RCC, and root C:N typically cluster on the same side of the PC1 axis (reflecting a conservative strategy), while SLA, LNC, SRA, SRL, RNC, and RPC cluster on the other side of the PC1 axis (reflecting an acquisitive strategy). Although PC1 explained only 33%–55% of the total trait variance across different analyses, which showed a multi‐trait PCA due to multiple independent ecological constraints, and the consistent loading patterns and significant separation of altitude groups along PC1 (Figure 5), confirming that this axis captures the elevational gradient. Furthermore, clustering and divergence along the altitude gradient are shown among different samples, indicating the existence of LES, RES, and WES along the altitude gradient for *P. massoniana* and understory shrubs in the *P. massoniana* plantations (Figures 5 and 6; Table S3), which is in line with the results of previous research (Ciccarelli et al. 2023; de la Riva et al. 2021; de la Riva et al. 2015; Fortunel et al. 2012; Freschet et al. 2010; Li et al. 2022; Yang, Xiao, et al. 2021; Zhang et al. 2024). The LES, RES, and WES of *P. massoniana* showed a shift from an acquisition strategy at low altitudes (larger SLA, SRA, and SRL; higher LNC, RNC, and RPC; smaller LT and RD; and lower LDMC) to a conservative strategy at high altitudes, which is consistent with the hypothesis that *P. massoniana* adapts to the potentially more stressful environment at high altitudes by increasing the traits (e.g., LDMC, LT, RD, RCC) to bear higher construction costs. Related research in both the same area and other areas indicates that with the increase in altitude, leaves, roots, and whole‐plants are more likely to adopt a conservative adaptation strategy (Bai et al. 2015; Freschet et al. 2010; Pierick et al. 2023; Yang, Xiao, et al. 2021; Zhu et al. 2022). Because resource conservation is a common strategy for plants to respond to environmental or resource stress (Wright et al. 2004; Wright and Westoby 2002). In contrast, the PES of understory shrubs exhibits a resource adaptation strategy contrary to that of *P. massoniana* . This difference is consistent with the niche differentiation and differences in resource utilization strategies of plants with different life forms along the same environmental gradient (Li et al. 2025). In addition, our study also found that there are also LES, RES, and WES in two woody plants with different life forms under the *P. massoniana* plantations. *P. massoniana* (tree) is located at the conservative end of the three economic spectrums, while the understory shrubs are all located at the acquisition end. This result is consistent with the research results of subtropical forests and other forest ecosystems (Du et al. 2024; Feng et al. 2022; Fyllas et al. 2020). This differentiation of resource allocation strategies along the altitude gradient further supports the applicability of the PES theory in subtropical forest ecosystems (de la Riva et al. 2021).

### 4.2 Elevation Trade‐Off and Consistency of Plants LES, RES and WES in Subtropical Mountain *P. massoniana* Plantations

Plants respond to changes in environmental gradients by modifying their resource utilization strategies, which is characterized by the trade‐offs and synergies among different traits within organs (Diaz et al. 2016; Reich 2014). We found that *P. massoniana* showed trade‐offs among traits at the leaf, root and whole‐plant levels to adapt to altitudinal changes (Figure 2a,d). As a species preferring warm temperatures and intolerant to cold, its growth, development and environmental responses are known to vary with mountain altitude gradients (Jing et al. 2022), and its distribution has an upper limit of approximately 1300 m in subtropical Southwest China (Zhou 2001). Consequently, *P. massoniana* likely adapts to high‐altitude habitats through multi‐trait synergy and trade‐offs. Our research indicates that its leaf, root, and whole‐plant traits collectively shift from a resource‐acquisitive strategy at low altitudes to a conservative strategy at high altitudes, supporting the hypothesis that its growth is affected by altitudinal stress. This aligns with other studies also suggest that plants tend to adopt a conservative resource utilization strategy to adapt to stressful habitats. For example, recent studies show that with the increase of the altitude gradient and the decrease of temperature, the functional trait space and redundancy of plant communities in Southwest China show a decreasing trend (Zhang et al. 2023). Similarly, Zhu et al. also shows that SLA, LNC and LPC of *P. massoniana* in subtropical forest ecosystems tend to decrease in high altitude habitats (Zhu et al. 2022). This shift from acquisition to conservation with altitude is mainly attributed to decreased temperature and altered water availability at higher elevations (Milla et al. 2009; Poorter et al. 2009).

In contrast, the understory shrubs in the *P. massoniana* plantations showed a consistent resource trade‐off strategy at the leaf, root, and whole‐plant levels that was opposite to *P. massoniana* (Figure 2d,e,h). This divergence likely stems from the shrubs' lower sensitivity to the relatively limited altitudinal gradient change (300 m in this study) and the predominant influence of other local environmental factors like canopy‐regulated microclimate and soil physicochemical properties. Although altitude gradients typically affect plant functional traits via macro‐environmental factors like temperature and precipitation (Körner 2021), in karst mountain ecosystems, the impact of extreme spatial heterogeneity in soil properties (e.g., pH, nutrient availability, moisture) on shrub resource acquisition may surpass macroclimatic effects (Luo et al. 2026; Wang et al. 2025). Specifically, soil nutrient availability in the study area may not follow a consistent altitudinal stress gradient and can even show localized enrichment, thereby weakening the direct driving effect of altitude on shrub resource trade‐offs. Furthermore, the niche complementarity hypothesis indicates that in order to reduce competition and utilize resources efficiently, species form niche complementarity in aspects such as resource acquisition and space utilization. When the niche occupied by a certain species decreases, other species will adjust their own ecological strategies to occupy the niche space vacated by this species (Hao et al. 2020). Therefore, when the resource competition pressure of *P. massoniana* changes due to factors such as altitude and its occupied niche changes accordingly, the understory shrubs may adjust their own resource trade‐off strategies from the leaf to the whole‐plant level correspondingly. For instance, they might adopt a conservative strategy at low altitudes and switch to an acquisitive strategy at high altitudes to achieve niche complementarity with *P. massoniana* . Additionally, inherent differences in niche breadth and plasticity between coniferous and broadleaved functional types (Liu et al. 2025; Zhang, Chen, et al. 2020), coupled with the potentially higher phenotypic plasticity of broadleaved shrubs—especially in response to understory light and soil nutrition (Bastias et al. 2018; Letts et al. 2012)—could allow shrubs to flexibly adjust to microenvironmental gradients shaped by canopy and soil changes rather than directly responding to the macroclimatic altitude gradient. This perspective is supported by studies highlighting the strong regulatory role of the *P. massoniana* plantations canopy, which redistributes light and water, shapes the local environment, and is a key factor affecting understory plant functional traits (Kermavnar et al. 2021a, 2021b; Pelt and Franklin 2000). For example, *P. massoniana* plantations canopy structure can significantly alter understory light and soil moisture, decoupling understory diversity distribution from altitude (Cui et al. 2014), and canopy variables are significant factors influencing shrub layer plant traits (Cheng et al. 2022). In summary, the invariant or inverse variation of understory shrub strategies along the altitude is likely a comprehensive manifestation of their response to multiple factors, including canopy‐regulated microclimate, local soil heterogeneity, and interspecific competition regimes, rather than a direct reaction to altitudinal climatic stress per se.

Our research also revealed significant coordination among traits of different plant organs, supporting the existence of a PES along the altitude gradient in the subtropical mountain forest ecosystem. Whether it is *P. massoniana* , understory shrubs, or all the woody plants in the *P. massoniana* plantations integrating *P. massoniana* and shrubs, there is a consistent resource utilization strategy across organs (Figure 7). This indicates that *P. massoniana* and understory shrubs form a cross‐organ functional modular integration through the systematic coordination of leaf and root traits (such as the coupling of high LDMC and low SRL) (Freschet et al. 2021), so as to maintain the balance between resource acquisition and conservation in habitats with different altitude gradients (e.g., decreasing temperature and increasing soil nutrient heterogeneity) (Candeias and Fraterrigo 2020; Körner 2021). A methodological caveat should be noted regarding root trait measurement: the visual‐olfactory separation of *P. massoniana* roots from shrub roots, while reliable in a pure plantation setting due to species‐specific resinous odor and morphology (Rewald et al. 2012), may still introduce minor identification errors that could affect community‐level root trait estimates. However, given the clear morphological differences and the fact that all non‐ *P. massoniana* roots were pooled as a single shrub community sample (using CWM), such errors are unlikely to bias the main conclusions about altitudinal trends. Leaves and roots, as the primary organs for plant nutrient absorption and growth, generally exhibit synergistic absorption and utilization of nutrients (Reich 2014). When leaves are in a state of rapid resource acquisition, roots also need to be in a state of rapid resource acquisition to obtain more nutrients and maintain the optimal growth state (Delpiano et al. 2020; Li et al. 2022; Reich 2014). Community level studies on subtropical forests also show that both leaf and root traits change synergistically along the resource acquisition‐conservation economic spectrum, and there is a significant positive correlation in chemical traits (Luo et al. 2023). With the change of environmental gradients, there is significant coordination between the traits of different organs of Mediterranean forest plants (de la Riva et al. 2015). Other studies also show that the SLA and SRL of plant communities increase synchronously with the increase of altitude along the altitude gradient, reflecting a systematic shift from resource conservative to resource acquisition strategies (Song et al. 2025). Moreover, coordination between leaf and root traits has also been observed in alpine coniferous plants, coastal dune plants, and even ferns (Ciccarelli et al. 2023; Li et al. 2022; Zhang et al. 2024). It suggests that the synergy among multiple organs may be a common pattern for plant environmental adaptation. In addition, we defined a coherent WES in PC1 around multiple co‐varying leaf and root traits (Figure 2g–i). This specialized axis is mainly constructed by the structural and chemical investment variables of plant vegetative organs, thus supporting to some extent the consistent resource trade‐off strategy of plants at the whole‐plant level (Ciccarelli et al. 2023). This resource economic spectrum is completely consistent with the current understanding of the influence of the environment on plant traits and life strategies (Ackerly and Cornwell 2007; Diaz et al. 1998; Freschet et al. 2010). At the same time, the clustering of life forms along this economic spectrum (Figure 2i) is also consistent with previous studies (Freschet et al. 2010). This result further confirms the connection with the established plant strategy scheme. Our study focused on describing the altitudinal patterns of PES, not on isolating causal environmental drivers. Thus, the observed associations are correlated with elevation. Future work should combine direct environmental measurements to disentangle the drivers of these patterns.

## 5 Conclusion

The PES illustrates the trade‐offs and coordination among multiple functional traits. Our study reveals linkages between leaf, root, and whole‐plant traits of woody plants in *P. massoniana* plantations and demonstrates the existence of LES, RES, and WES along an altitudinal gradient. With increasing altitude, *P. massoniana* exhibits a consistent ecological strategy shift from acquisitive to conservative patterns across leaves, roots, and the whole plant, indicating that altitudinal variation is associated with changes in its resource utilization strategy. In contrast, understory shrubs show opposite or invariant patterns, which may be primarily shaped by local microenvironmental factors like canopy structure and soil heterogeneity rather than direct altitudinal climatic stress. Consequently, forest management practices should be tailored to specific altitude zones. For instance, at lower altitudes where *P. massoniana* exhibits acquisitive traits, thinning could focus on reducing intraspecific competition to optimize resource use. At middle to high altitudes, where the trees adopt a conservative strategy and shrubs show contrasting acquisitive trends, management should promote the regeneration and maintenance of a diverse understory shrub layer to enhance overall community resilience and resource partitioning. However, several caveats should be noted. The observed altitudinal gradient spans only 300 m (1200–1500 m a.s.l.), representing a restricted elevation range; whether the same patterns hold across broader gradients remains to be tested. Given the observational nature of this study, the reported altitudinal trends and correlations do not imply causation, and further analysis integrating environmental factors such as temperature, soil nutrients, and light availability is needed to identify the drivers of the observed trait shifts. Within these limitations, this study provides empirical evidence for the applicability of the PES theory for understanding plant adaptation mechanisms in the fragile habitats of the subtropical mountain forest ecosystem, and offers a theoretical basis for predicting the successional direction of subtropical mountain forests under climate change and for optimizing forest management.

## Author Contributions

**Kun Nie:** data curation (lead), investigation (lead), visualization (lead), writing – original draft (lead). **Ming Xu:** funding acquisition (equal), methodology (equal), writing – review and editing (equal). **Jian Zhang:** conceptualization (lead), funding acquisition (equal), investigation (equal), methodology (lead), supervision (equal), writing – original draft (supporting), writing – review and editing (lead).

## Funding

This work was supported by the National Nature Science Foundation of China (NSFC) project (31960234 and 31660150).

## Conflicts of Interest

The authors declare no conflicts of interest.

## Supporting information


**Table S1:** Information of sampling sites and plots.
**Table S2:** Sampled dominant understory shrub species for trait measurements, with their frequency of occurrence and importance values.
**Table S3:** Bivariate relationships between individual traits and leaf, root, whole‐plant economic spectrum characteristics of plants in *P. massoniana* plantations at different altitudes and the scores of PC1 and PC2.
**Figure S1:** Species composition and importance values of understory shrub species in *Pinus massoniana* plantations.
**Figure S2:** Detailed shrub sampling information for each sampling plot.
**Figure S3:** Responses of plant functional traits of *P. massoniana* and understory shrubs to altitude gradients at different altitude. The coral red circles and lines respectively represent the functional traits of *P. massoniana* and the univariate regression lines between these traits and altitude, while the cerulean blue circles and lines respectively represent the functional traits of shrubs and the univariate regression lines between their traits and altitude. *y*_*pm*_ denotes the simple linear regression equation for the functional traits of *P. massoniana* , while *y*_*shrub*_ stands for the simple linear regression equation for the functional traits of understory shrubs (***, *p* ≤ 0.001; **, *p* ≤ 0.01; *, *p* ≤ 0.05; ns, *p* > 0.05).
**Figure S4:** The multiple factor analysis (MFA) on 18 functional traits. Cerulean blue represents leaf traits. Coral red represents root traits.
**Figure S5:** Contribution rates of plant functional traits to axes PC1 and PC2. (a) Leaf traits of *Pinus massoniana* ; (b) Leaf traits of understory shrubs; (c) Leaf traits of woody plants ( *P. massoniana* and understory shrubs); (d) Root traits of *P. massoniana* ; (e) Root traits of understory shrubs; (f) Root traits of woody plants; (g) Whole‐plant leaf traits of *P. massoniana* ; (h) Whole‐plant traits of understory shrubs; (i) Contribution rates of whole‐plant traits of woody plants to axes PC1 and PC2. The red dashed line in the figure represents the expected average contribution. Variables greater than this value are considered to make significant contributions to the principal component.

## Data Availability

Data will be made available on request.
