# Supplementary material for: Altitudinal Variation in Trade‐Off Strategies of the Plant Economics Spectrum of Pinus massoniana Plantations in Subtropical Mountains
Source: Ecol Evol. 2026 Jul 10;16(7):e73977. doi: 10.1002/ece3.73977 (PMC13354548; doi:10.1002/ece3.73977)
Supplement: Supplementary file 1 — Table S1: Information of sampling sites and plots. Table S2: Sampled dominant understory shrub species for trait measurements, with their frequency of occurrence and importance values. Table S3: Bivariate relationships between individual traits and leaf, root, whole‐plant economic spectrum characteristics of plants in P. massoniana plantations at different altitudes and the scores of PC1 and PC2. Figure S1: Species composition and importance values of understory shrub species in Pinus massoniana plantations. Figure S2: Detailed shrub sampling information for each sampling plot. Figure S3: Responses of plant functional traits of P. massoniana and understory shrubs to altitude gradients at different altitude. The coral red circles and lines respectively represent the functional traits of P. massoniana and the univariate regression lines between these traits and altitude, while the cerulean blue circles and lines respectively represent the functional traits of shrubs and the univariate regression lines between their traits and altitude. y pm denotes the simple linear regression equation for the functional traits of P. massoniana , while y shrub stands for the simple linear regression equation for the functional traits of understory shrubs (***, p ≤ 0.001; **, p ≤ 0.01; *, p ≤ 0.05; ns, p > 0.05). Figure S4: The multiple factor analysis (MFA) on 18 functional traits. Cerulean blue represents leaf traits. Coral red represents root traits. Figure S5: Contribution rates of plant functional traits to axes PC1 and PC2. (a) Leaf traits of Pinus massoniana ; (b) Leaf traits of understory shrubs; (c) Leaf traits of woody plants ( P. massoniana and understory shrubs); (d) Root traits of P. massoniana ; (e) Root traits of understory shrubs; (f) Root traits of woody plants; (g) Whole‐plant leaf traits of P. massoniana ; (h) Whole‐plant traits of understory shrubs; (i) Contribution rates of whole‐plant traits of woody plants to axes PC1 and PC2. The red dashed line in the figure re [file ECE3-16-e73977-s001.docx]

**Supplementary information**

**Altitudinal variation in trade-off strategies of the plant economics spectrum of *Pinus massoniana* plantations in subtropical mountains**

Kun Nie^1,2^, Ming Xu^1^, Jian Zhang^1*^

*^1^ College of Life Sciences, Guizhou Key Laboratory of Agricultural Microbiology, Guizhou University, Guiyang 550025, China.*

*^2^ National Key Laboratory of Green Pesticide, Key Laboratory of Green Pesticide and Agricultural Bioengineering, Ministry of Education, Center for R&D of Fine Chemicals of Guizhou University, Guiyang 550025, China*

^*^ Correspondence:

Ming Xu, College of Life Sciences, Guizhou University, Guiyang 550025, China. Email: mingxu566@163.com

Jian Zhang, College of Life Sciences, Guizhou University, Guiyang 550025, China.

Email: zhangjian12102@163.com

**Table S1** Information of sampling sites and plots.

| Plot | Altitude (m a.s.l) | Slope (°) | Age of forest (a) | Average diameter at breast height of *P. massoniana* (cm) | Average height of *P. massoniana* (m) | Canopy Density (%) | Soil Type |
| --- | --- | --- | --- | --- | --- | --- | --- |
| 1200-1 | 1200 | 25 | 41 | 40.00 | 13.63 | 53 | Calcareous soil |
| 1200-2 | 1202 | 35 | 40 | 34.46 | 13.87 | 81 | Calcareous soil |
| 1200-3 | 1204 | 30 | 42 | 44.56 | 15.57 | 84 | Calcareous soil |
| 1300-1 | 1303 | 18 | 53 | 65.71 | 17.25 | 77 | Calcareous soil |
| 1300-2 | 1308 | 9 | 52 | 62.04 | 16.70 | 72 | Calcareous soil |
| 1300-3 | 1300 | 32 | 52 | 72.40 | 16.03 | 78 | Calcareous soil |
| 1400-1 | 1401 | 15 | 50 | 73.14 | 19.00 | 76 | Calcareous soil |
| 1400-2 | 1409 | 4 | 51 | 53.12 | 19.07 | 74 | Calcareous soil |
| 1400-3 | 1404 | 6 | 50 | 62.35 | 17.98 | 77 | Calcareous soil |
| 1500-1 | 1504 | 12 | 52 | 57.08 | 16.02 | 71 | Calcareous soil |
| 1500-2 | 1506 | 14 | 48 | 56.63 | 15.96 | 72 | Calcareous soil |
| 1500-3 | 1500 | 7 | 45 | 53.47 | 15.68 | 68 | Calcareous soil |

Mean DBH: mean diameter at breast high; Mean TH: mean tree’s height.

**Table S2** Sampled dominant understory shrub species for trait measurements, with their frequency of occurrence and importance values.

| Sampled species | Number and frequency of occurrence in all quadrats | Mean of importance value |
| --- | --- | --- |

| *Rhododendron simsii* | 53 (0.88%) | 23.37 |
| --- | --- | --- |
| *Quercus dentata* | 49 (0.82%) | 17.04 |
| *Smilax polycolea* | 44 (0.73%) | 5.54 |
| *Castanea seguinii* | 43 (0.72%) | 12.49 |
| *Eurya japonica* | 34 (0.57%) | 8.78 |
| *Clethra delavayi* | 24 (0.40%) | 17.56 |
| *Rubus corchorifolius* | 20 (0.33%) | 13.70 |

Note: Five shrub survey quadrats were established within each sampling plot, resulting in a total of 60 quadrats.

**Table S3** Bivariate relationships between individual traits and leaf, root, whole-plant economic spectrum characteristics of plants in *P. massoniana* plantations at different altitudes and the scores of PC1 and PC2.

|  | Leaf economics spectrum | Root economics spectrum | Whole-plant economics spectrum |
| --- | --- | --- | --- |

|  | PM | PM | Shrub | Shrub | All | All | PM | PM | Shrub | Shrub | All | All | PM | PM | Shrub | Shrub | All | All |
| --- | --- | --- | --- | --- | --- | --- | --- | --- | --- | --- | --- | --- | --- | --- | --- | --- | --- | --- |
|  | PC1 | PC2 | PC1 | PC2 | PC1 | PC2 | PC1 | PC2 | PC1 | PC2 | PC1 | PC2 | PC1 | PC2 | PC1 | PC2 | PC1 | PC2 |
| LCC | 0.479 *** | 0.128 ns | 0.027 ns | 0.791 *** | -0.83*** | -0.288** |  |  |  |  |  |  | 0.45 *** | 0.085 ns | 0.027 ns | 0.343 * | -0.776*** | 0.148ns |
| LNC | 0.9 *** | -0.08 ns | 0.895 *** | 0.357 * | 0.977*** | -0.074ns |  |  |  |  |  |  | 0.795 *** | -0.085 ns | 0.792 *** | 0.541 *** | 0.945*** | -0.171ns |
| LPC | -0.41 *** | -0.752 *** | 0.906 *** | -0.183 ns | 0.778*** | 0.564*** |  |  |  |  |  |  | -0.368 ** | -0.402 *** | 0.878 *** | 0.024 ns | 0.747*** | -0.032ns |
| LKC | -0.372 ** | 0.714 *** | -0.801 *** | -0.34 * | 0.853*** | -0.031ns |  |  |  |  |  |  | -0.455 *** | 0.55 *** | -0.845 *** | -0.068 ns | 0.794*** | -0.268** |
| Leaf C:N | -0.837 *** | 0.124 ns | -0.89 *** | -0.025 ns | -0.935*** | 0.227* |  |  |  |  |  |  | -0.733 *** | 0.118 ns | -0.786 *** | -0.4 * | -0.904*** | 0.159ns |
| Leaf N:P | 0.91 *** | 0.266 * | -0.329 * | 0.666 *** | 0.765*** | -0.627*** |  |  |  |  |  |  | 0.807 *** | 0.108 ns | -0.403 * | 0.569 *** | 0.75*** | -0.208* |
| LDMC | -0.671 *** | -0.2 ns | -0.909 *** | 0.163 ns | -0.856*** | 0.103ns |  |  |  |  |  |  | -0.634 *** | -0.184 ns | -0.871 *** | -0.178 ns | -0.844*** | 0.024ns |
| LT | -0.614 *** | 0.465 *** | 0.413 * | -0.418 * | -0.962*** | -0.02ns |  |  |  |  |  |  | -0.646 *** | 0.214 ns | 0.434 ** | -0.12 ns | -0.934*** | 0.18ns |
| SLA | 0.806 *** | -0.041 ns | 0.96 *** | 0.1 ns | 0.902*** | 0.202* |  |  |  |  |  |  | 0.791 *** | 0.067 ns | 0.908 *** | 0.287 ns | 0.891*** | -0.037ns |
| RCC |  |  |  |  |  |  | -0.292 ns | -0.122 ns | -0.285 ns | 0.043 ns | -0.649*** | 0.089ns | -0.127 ns | -0.112 ns | -0.338 * | 0.191 ns | -0.677*** | -0.014ns |
| RNC |  |  |  |  |  |  | 0.789 *** | -0.56 *** | 0.577 *** | 0.794 *** | 0.793*** | -0.469*** | 0.657 *** | -0.339 ** | 0.476 ** | -0.001 ns | 0.743*** | -0.108ns |
| RPC |  |  |  |  |  |  | 0.668 *** | -0.12 ns | 0.767 *** | 0.013 ns | 0.755*** | 0.215* | 0.757 *** | -0.124 ns | 0.732 *** | -0.409 * | 0.668*** | 0.456*** |
| RKC |  |  |  |  |  |  | 0.787 *** | -0.127 ns | 0.226 ns | -0.243 ns | 0.697*** | -0.205* | 0.763 *** | 0.031 ns | 0.24 ns | -0.22 ns | 0.653*** | 0.044ns |
| Root C:N |  |  |  |  |  |  | -0.773 *** | 0.554 *** | -0.632 *** | -0.743 *** | -0.803*** | 0.527*** | -0.598 *** | 0.332 ** | -0.551 *** | 0.058 ns | -0.761*** | 0.17ns |
| Root N:P |  |  |  |  |  |  | 0.063 ns | -0.426 ** | -0.476 ** | 0.474 ** | -0.09ns | -0.794*** | -0.163 ns | -0.203 ns | -0.499 ** | 0.443 ** | 0.004ns | -0.731*** |
| RD |  |  |  |  |  |  | -0.599 *** | -0.563 *** | -0.527 *** | 0.45 ** | -0.751*** | -0.132ns | -0.307 ** | -0.76 *** | 0.057 ns | 0.775 *** | -0.638*** | -0.32*** |
| SRL |  |  |  |  |  |  | 0.547 *** | 0.811 *** | 0.781 *** | -0.394 * | 0.807*** | 0.381*** | 0.295 * | 0.866 *** | 0.083 ns | -0.896 *** | 0.671*** | 0.558*** |
| SRA |  |  |  |  |  |  | 0.461 ** | 0.824 *** | 0.713 *** | -0.239 ns | 0.663*** | 0.536*** | 0.279 * | 0.801 *** | 0.026 ns | -0.771 *** | 0.468*** | 0.696*** |

Note: Significant correlations are presented in boldface (***, *P* ≤ 0.001; **, *P* ≤ 0.01; *, *P* ≤ 0.05; ns, *P* > 0.05).


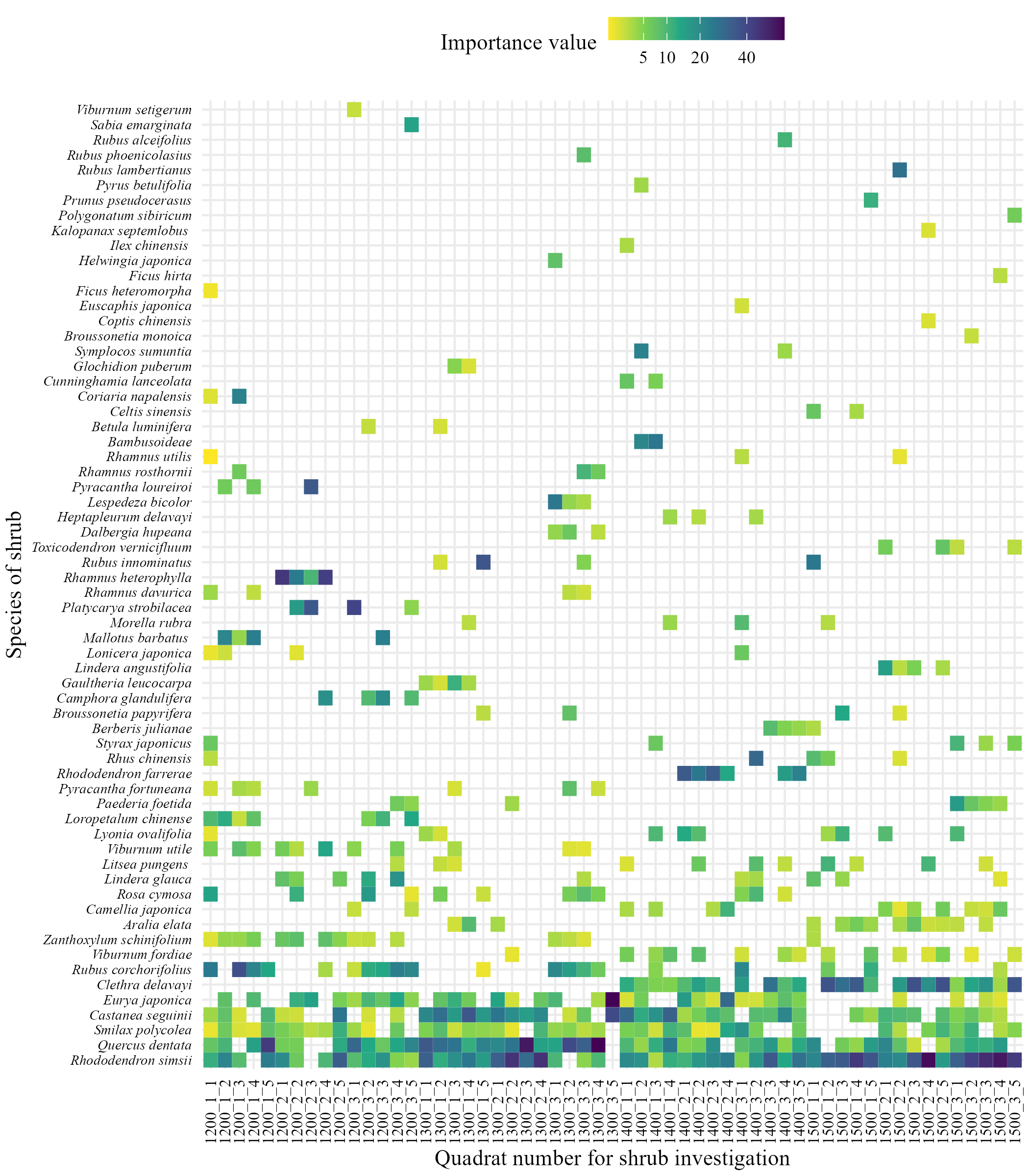


**Figure S1** Species composition and importance values of understory shrub species in *Pinus massoniana* plantations.


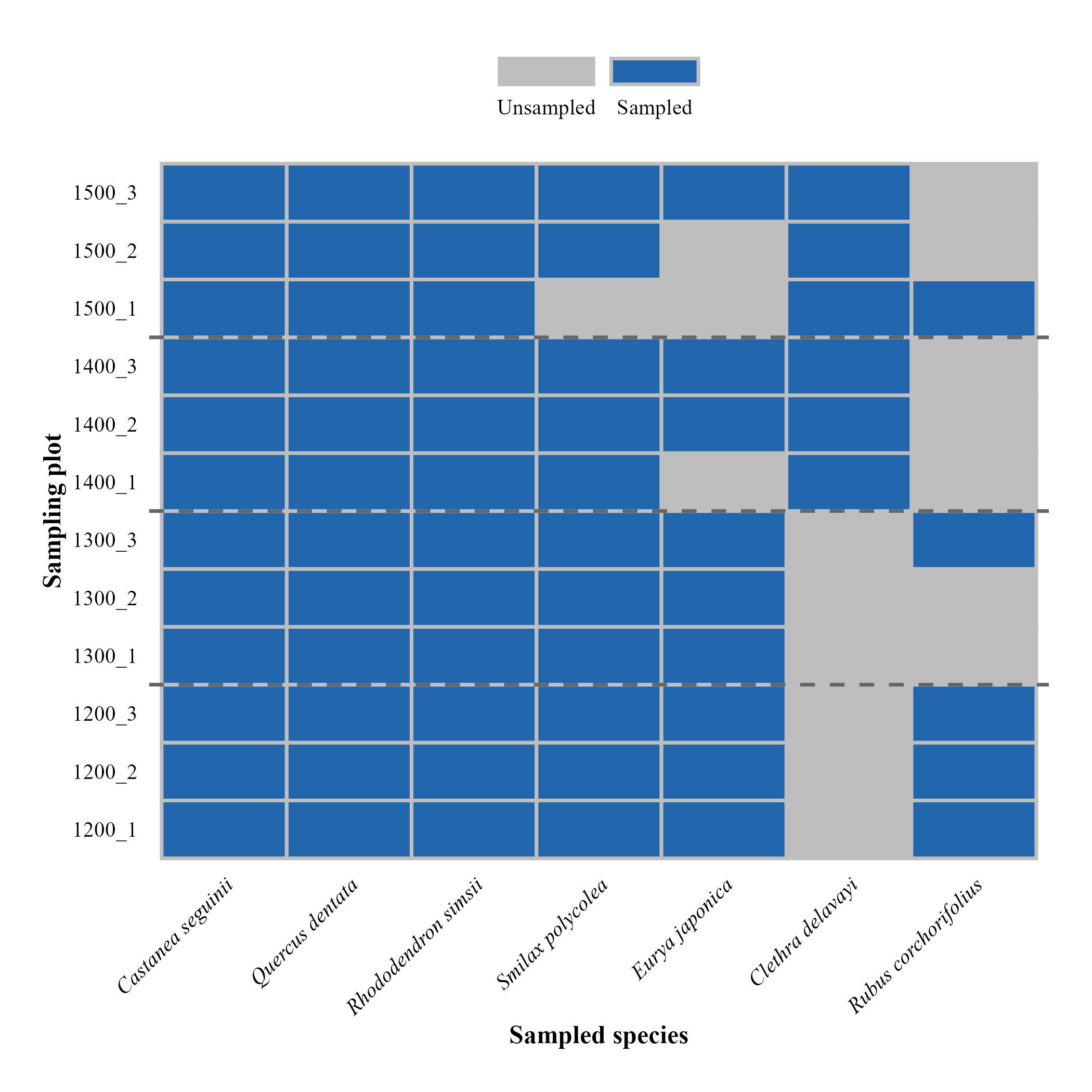


**Figure S2** Detailed shrub sampling information for each sampling plot.





**Figure S3** Responses of plant functional traits of *P. massoniana* and understory shrubs to altitude gradients at different altitude. The coral red circles and lines respectively represent the functional traits of *P. massoniana* and the univariate regression lines between these traits and altitude, while the cerulean blue circles and lines respectively represent the functional traits of shrubs and the univariate regression lines between their traits and altitude. *y_pm_* denotes the simple linear regression equation for the functional traits of *P. massoniana*, while *y_shrub_* stands for the simple linear regression equation for the functional traits of understory shrubs (***, *P* ≤ 0.001; **, *P* ≤ 0.01; *, *P* ≤ 0.05; ns, *P* > 0.05).


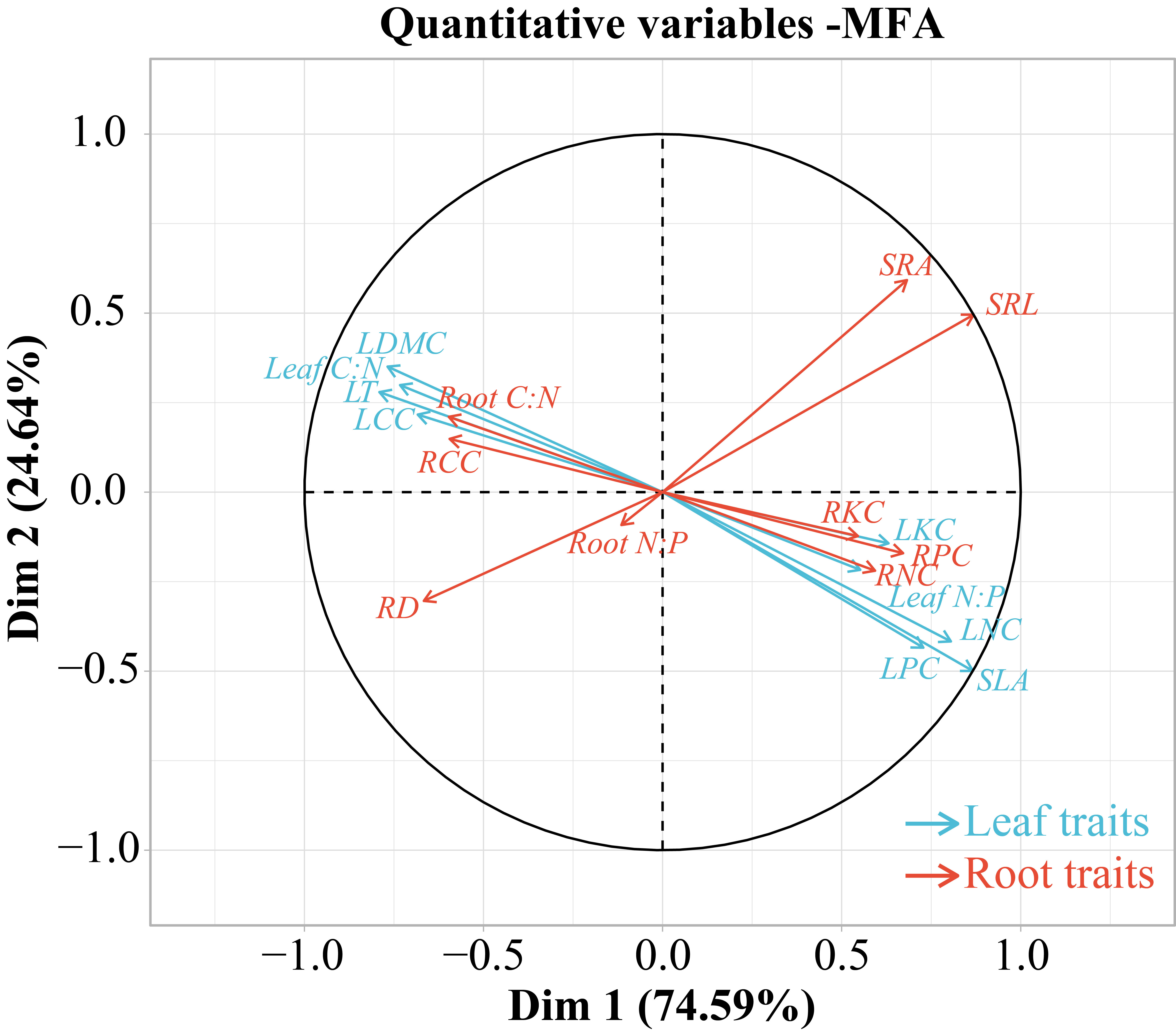


**Figure S4** The multiple factor analysis (MFA) on 18 functional traits. Cerulean blue represents leaf traits. Coral red represents root traits.





**Figure S5** Contribution rates of plant functional traits to axes PC1 and PC2.

(a) Leaf traits of *Pinus massoniana*; (b) Leaf traits of understory shrubs; (c) Leaf traits of woody plants (*P. massoniana* and understory shrubs); (d) Root traits of *P. massoniana*; (e) Root traits of understory shrubs; (f) Root traits of woody plants; (g) Whole-plant leaf traits of *P. massoniana*; (h) Whole-plant traits of understory shrubs; (i) Contribution rates of whole-plant traits of woody plants to axes PC1 and PC2. The red dashed line in the figure represents the expected average contribution. Variables greater than this value are considered to make significant contributions to the principal component.
